# Radiation dose in CT-based active surveillance of small renal masses may be reduced by 75%: A retrospective exploratory multiobserver study

**DOI:** 10.1016/j.redii.2022.100019

**Published:** 2022-12-13

**Authors:** Jens Borgbjerg, Nis Elbrønd Larsen, Ivar Mjåland Salte, Niklas Revold Grønli, Elise Klæstrup, Anne Negård

**Affiliations:** a Department of Radiology, Akershus University Hospital, Sykehusveien 25, Nordbyhagen 1478, Norway; b Department of Radiology, Aarhus University Hospital, Aarhus, Denmark

## 1 Introduction

Active surveillance is increasingly utilized in managing small renal masses (SRMs, i.e. diameter < 4 cm corresponding to stage cT1a) regardless of patient age [1]. The most common threshold metrics prompting cross-over from an active surveillance protocol to delayed intervention are linear tumor growth rate (maximum axial diameter > 0.5 cm/year) and size > 4 cm^2^.

CT is the imaging modality used most frequently [1] and is usually more precise than ultrasound and more accessible than MRI. However, the cumulative radiation dose incurred in serial imaging as part of active surveillance is a concern [3]. We hypothesized that contrast-enhanced low-dose CT with a 75% reduction in radiation dose could be used interchangeably with normal-dose CT in terms of accuracy and reproducibility in determining the maximum diameter of SRMs.

## 2 Methods

We included 40 CTs from the 2019 Kidney and Kidney Tumor Segmentation Challenge, where patients underwent a preoperative abdominal CT for a renal mass [4] (Table 1). Inclusion criteria were: tumor size < 5 cm, intravenous contrast enhancement in the late-arterial phase, and non-infiltrating tumor. To generate simulated low-dose CT (LDCT) images corresponding to a 75% radiation dose reduction, we used the technique utilized by Juluru et al. [5], in which spatially correlated noise obtained from scanning a phantom is introduced into reference normal-dose CT datasets (NDCT). This method assumes that reconstruction techniques of reference datasets conserve a Poisson distribution of noise which is the case with filtered back-projection and Sinogram Affirmed Iterative Reconstruction (Table 1). The added noise was based on a soft tissue kernel (B40) of the Siemens Somatom Definition AS 64 system (Siemens, Erlangen, Germany). Resident (*n* = 2) and consultant (*n* = 4) radiologists from two university hospitals independently performed blinded measurements of the NDCT and LDCT cases in a two-session mixed-order setup using a web-based DICOM viewer[6]. There was a 10-day interval between sessions. Orthogonal multiplanar reconstruction capability was available to help locate and measure maximal axial diameter, with windowing and leveling performed at the discretion of each observer. For each case, observers rated diagnostic confidence in delineating the contour of the renal mass on a five-point scale (1, poor; 2, fair; 3, good; 4, very good; and 5, excellent).

**Table 1:** Demographics data, CT and Tumor Characteristics of study patients (*n* = 40).

Patients	
Mean age (y)	58.9 (+/−14.8, 12–81)
Sex (female/male)	20 (50%) / 20 (50%)
Mean BMI (kg/m^2^)	30.2 (+/−6.6, 16.2–45.2)
Tumor characteristics	
Mean tumor size (cm)	2.4 (+/−0.82, 1.2–4.5)
Tumor histology	
Clear cell RCC	26 (65%)
Papillary RCC	5 (13%)
Oncocytoma	4 (10%)
Chromophobe RCC	1 (3%)
Angiomyolipoma	2 (5%)
Multilocular cystic RCC	1 (3%)
Spindle cell neoplasm	1 (3%)
CT characteristics	
No. different CT systems	17
Image reconstruction	
Filtered back-projection	26 (65%)
SAFARI iterative reconstruction	14 (35%)
Mean scan length (cm)^a^	29.4 (+/−9.8, 20.0–60.0)
Mean tube voltage (kVp)	112.3 (+/−10.4, 1–5)
Mean tube current (mAs)	352.8 (+/−104.9, 169.8–558.6)
Mean slice thickness (mm)	3.8 (+/−1.4, 1–5)
Mean CTDI_vol_ (mGy)^b^	12.6 (+/−8.1, 4.5–39.2)
Mean DLP (mGy x cm)^b^	342.1 (+/−188.4, 121.9–917.3)
Mean effective dose (mSv)^b^^,^^c^	5.1 (+/−2.8, 1.8–13.8)
Mean noise (NDCT, HU)^d^	17.1 (+/- 6.3, 8–32)
Mean noise (LDCT, HU)^d^	34.2 (+/- 12.6, 16–64)

Unless otherwise indicated, data are numbers and data in parentheses are percentages.

Mean data are presented with standard deviation and range in parentheses.

a From the diaphragm to the abdominal aortic bifurcation.

b Data available for 22 out of 40 CT scans.

c Effective dose = DLP x abdominal weighting factor (=0.015 mSv x mGy^−1^ x cm^−1^).

d Obtained from a region of interest placed in the abdominal aortic lumen at the level of the renal tumor evaluated. BMI, body mass index; RCC, renal cell carcinoma; DLP, dose length product; CTDIvol, CT dose index-volume; HU, Hounsfield unit; LDCT, low-dose CT; NDCT, normal-dose CT; SAFIRE, Sinogram Affirmed Iterative Reconstruction.

Measurement agreement was evaluated with the limits of agreement with the mean (LOAM), representing how much an observer's measurement may plausibly deviate from the mean of all observers’ measurements on the specific subject (i.e., a measure of reproducibility) [7]. In addition, we calculated Bland-Altman limits of agreement (LoA) intraobserver pairs of each observer for NDCT vs. LDCT.

## 3 Results

The mean diameter was 24.3 (±1.3) mm for NDCT and 24.2 (±1.3) mm for LDCT, yielding a non-significant mean difference of 0.1 mm (95% CI −0.1–0.3, *p* > 0.05). The 95% LOAM [CI] was similar for the NDCT and LDCT (2.7 [2.5–3.2] vs. 2.8 [2.6–3.9] mm).

**Fig. 1:**
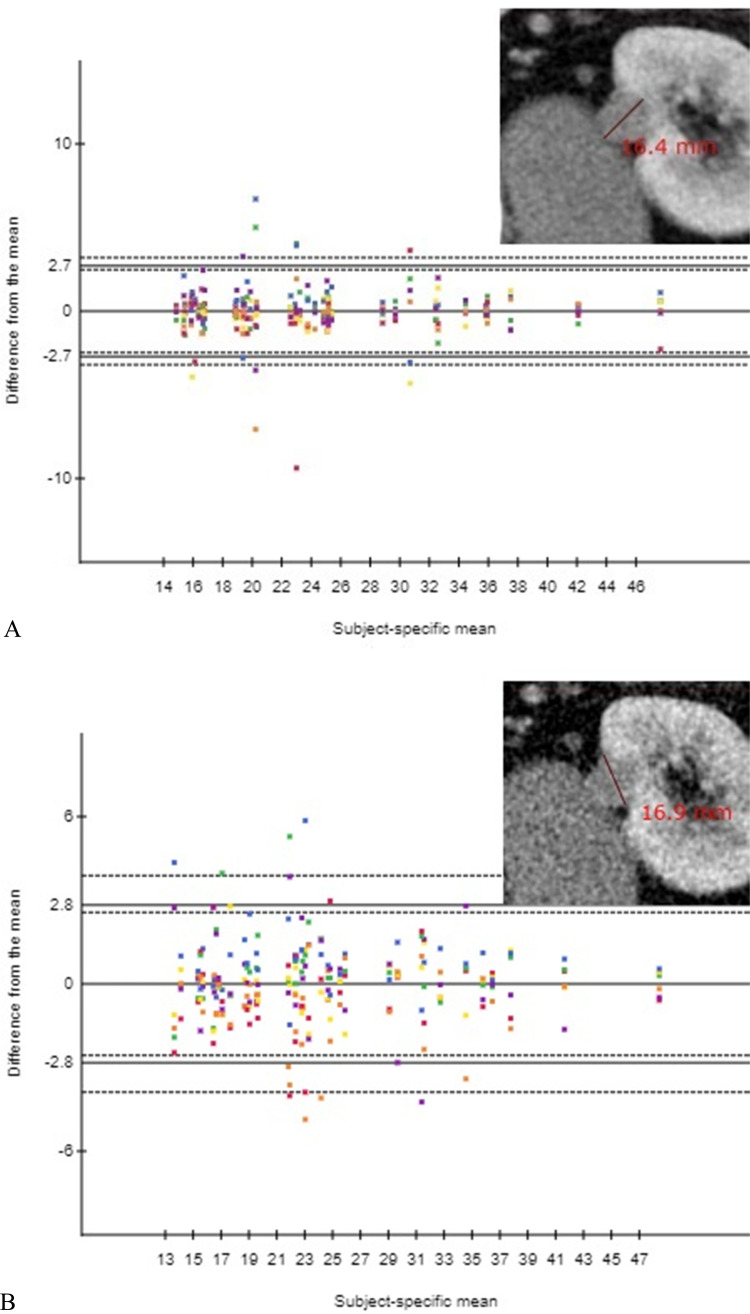
Observer agreement plots for measurements of maximal axial renal tumor diameter of the 480 measurements in (A) normal-dose CT and (B) simulated low-dose CT, respectively (horizontal axes represent the tumor-specific mean diameter measurements (mm), vertical axes represent the difference from the individual renal tumor diameter measurements to the tumor-specific mean (mm). Differently colored dots represent individual measurements of the six observers. Some dots have been superimposed. Horizontal solid lines indicate upper and lower 95% limits of agreement with the mean and a line of zero difference. Dashed lines correspond to the 95% confidence intervals for the limits of agreement). Screenshot of a sample tumor measurement shown in the upper right corner of each plot.

Agreement plots show that no observer systematically made unusual small or large measurements, giving no indication of heteroscedasticity associated with size (Fig. 1).

The observed measurement outliers outside the LOAM with accompanying CI for NDCT and LDCT were found to be in relation to >50% endophytic tumors having partly indistinct demarcation to the surrounding tissue.

The intraobserver (intermodality) LoA concerning NDCT vs. LDCT pairs (*n* = 6) yielded a mean LoA width of +/−3.6 mm. The diagnostic confidence of NDCT vs. LDCT was 3.7 and 3.1, mean difference of 0.6 (95% CI 0.5–0.7, *p* < 0.001). Measurement key images are available for review in a data repository [8].

## 4 Discussion

The results from this multi-observer study suggest NDCT and LDCT can be used interchangeably for size assessment in SRMs. Similar interobserver agreements for NDCT have been reported in the literature [2]. Even though the need to explore LDCT concerning active surveillance of SRMs was highlighted more than a decade ago [9], we know of no such prior studies.

Our results with substantial dose reduction in the range demonstrated feasible for urolithiasis evaluation corroborate the findings by Fletcher and colleagues [10]. They found an untapped potential for substantial dose reduction using existing CT technology across specific diagnostic tasks and that subjective image quality assessment declined before objective measures of observer performance. For example, in LDCT (40% dose reduction) reconstructed with filtered back-projection vs. NDCT, Fletcher et al. did not find inadequate performance in terms of reader agreement in the detection of hepatic metastases, whereas a 0.6 difference in subjective image quality using a five-point scale was observed [10].

A limitation of the current study is the noise simulation based on a single noise kernel which does not include possible noise variation due to different electron densities in different tissues. However, the simulated images do exhibit an image quality degradation in terms of noise level comparable to non-simulated CT images obtained at the corresponding lower tube current. Moreover, renal tumor morphology (i.e., attenuation and contour) was not evaluated, which in addition to tumor size, plays a role in the management recommendations by the American College of Radiology [1]. Results need cautious interpretation but should inform further research to translate results into clinical practice. Future studies should encompass larger, more diverse patient cohorts using either more precisely simulated LDCT derived from raw projection data or, ideally, prospectively acquired non-simulated LDCT.

## Authorship

All authors attest that they meet the current International Committee of Medical Journal Editors (ICMJE) criteria for Authorship and have made contributions to the paper as specified below.

1. Conception and design of the study

2. Acquisition of data

3. Analysis and interpretation of data

4. Drafting the article

5. Revising the article

6. Final approval

Jens Borgbjerg [1,3,4,5,6]

Nis Elbrønd Larsen [1,2,3,4,5,6]

Ivar Mjåland Salte [2,3,4,5,6]

Niklas Revold Grønli [2,3,4,5,6]

Elise Klæstrup [2,3,4,5,6]

Anne Negård [1,3,4,5,6]

## Human rights

The authors declare that the work described has been carried out in accordance with the Declaration of Helsinki of the World Medical Association revised in 2013 for experiments involving humans.

## Informed consent and patient details

The authors declare that this report does not contain any personal information that could lead to the identification of the patients.

## Funding

This work did not receive any grant from funding agencies in the public, commercial, or not-for-profit sectors.

## Declarations of Competing Interest

The authors declare that they have no conflict of interest and that they have full control of all primary data and that they agree to allow the journal to review their data if requested.
